# How far are we from the era of big data in transcriptomics? Lessons from the bacterial data in GEO

**DOI:** 10.1093/bib/bbaf560

**Published:** 2025-10-23

**Authors:** A S Escobedo-Muñoz, Diego Carmona-Campos, Armando G G Trapaga, Julio A Freyre-González

**Affiliations:** Regulatory Systems Biology Research Group, Program of Systems Biology; Undergraduate Program in Genomic Sciences, Center for Genomic Sciences, Universidad Nacional Autónoma de México, Av. Universidad s/n, Col. Chamilpa, Cuernavaca, Morelos 62210, México; Regulatory Systems Biology Research Group, Program of Systems Biology; Undergraduate Program in Genomic Sciences, Center for Genomic Sciences, Universidad Nacional Autónoma de México, Av. Universidad s/n, Col. Chamilpa, Cuernavaca, Morelos 62210, México; Regulatory Systems Biology Research Group, Program of Systems Biology; Undergraduate Program in Genomic Sciences, Center for Genomic Sciences, Universidad Nacional Autónoma de México, Av. Universidad s/n, Col. Chamilpa, Cuernavaca, Morelos 62210, México; Regulatory Systems Biology Research Group, Program of Systems Biology

**Keywords:** microarrays, RNA-seq, GEO, FAIR, metadata, normalization

## Abstract

The Gene Expression Omnibus (GEO) is the largest functional genomics repository, including ~5 million entries related to the main transcriptomic technologies: microarrays and RNA-seq. This amount of data has the potential to be reused in large-scale meta-analysis, such as those in bacterial systems biology, where the landscape of biological conditions is wider and more diverse than any individual experiment alone. Notwithstanding the accelerated growth in RNA-seq experiments, microarray still accounts for ~48% of bacterial transcriptomic entries in GEO, highlighting the need to revalue this data. Therefore, in this work, we assess the current state of bacterial microarray and RNA-seq data and metadata. We report diverse inconsistencies in both the GEO metadata documentation and community usage, limiting the automated access to biological context essential for high-throughput analysis interpretation. Additionally, while access to and analysis of RNA-seq data are topics widely discussed by the community, microarray data processing and normalization present challenges that need to be addressed for the proper data integration into large-scale reanalysis. Thus, we delve into the availability and processability of bacterial microarray data in GEO, showing a complex panorama where the lack of standard formats limits our reusability potential to at least 44% of the ~45 000 microarray entries. We conclude that GEO transcriptomic data and metadata should be viewed as valuable resources that require ongoing revision and maintenance. Finally, we propose a series of guidelines to enhance the Findability, Accessibility, Interoperability, and Reusability of GEO, thereby taking a step forward into the era of big data.

## Background

The current century has seen the rise of a new era whose protagonist is information, and its analysis is a priority in any field of human interest. Because of new technologies and their rapid evolution, we find that to obtain valuable conclusions from data, we must contend with a massive scale in terms of volume, velocity, and variety: big data [1].

**Figure 1:**
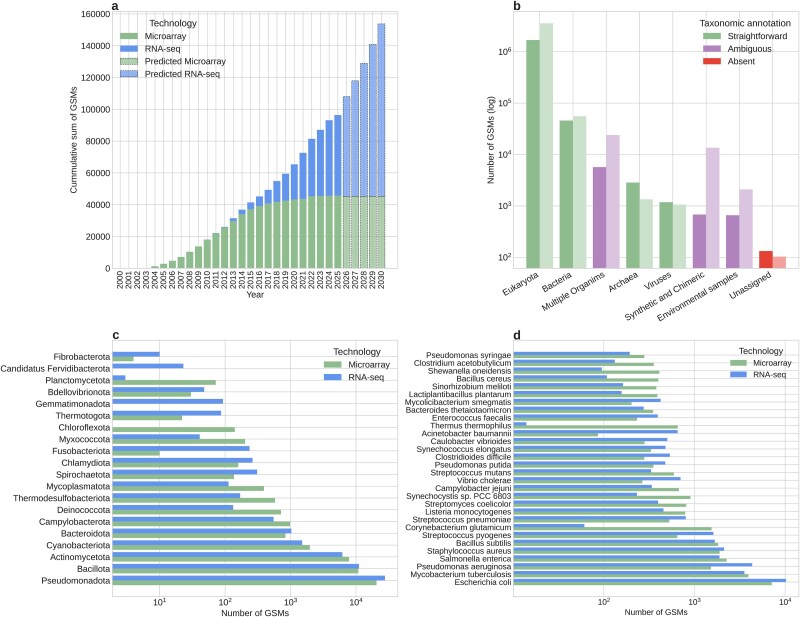
The amount of bacterial transcriptomic data available in GEO and the taxonomic bias. (a) The cumulative sum of the number of bacteria transcriptomic records submitted in GEO from 2000 to May 2025. “Expression profiling by array” in green, “Expression profiling by high-throughput sequencing” in blue. Using a least squares regression, we fitted the microarray data to a logistic function (${R}^2=0.999$, $y=45293.79/\left(1+{e}^{-0.3968\cdot\, \left(x-2011.245\right)}\right)$) and the RNA-seq data to a third-grade polynomial (${R}^2=0.992$, $y=7.25{x}^3-43578.00{x}^2+87367474.23x-58386137342.10$). We predict the number of records will increase by >1.6-fold by 2030 (light-colored dotted bars). (b) Distribution of the number of GSM entries of “expression profiling by array” (dark bar on the left) and “expression profiling by high-throughput sequencing” (light green on the right) technologies available in GEO. The entries are classified according to a biologically relevant taxonomic label generated from the superkingdom and domain of the reported organism in the GSM. The multiple organism category corresponds to GSMs annotated with more than one organism. Note that the y-axis is in a log_10_ scale. (c) Distribution of the number of GSMs corresponding to bacteria submitted in GEO grouped by the phylum of the annotated organism. (d) Distribution of the number of GSMs available in GEO for the 30 most represented bacteria (by genus and species). In (c) and (d), green bars correspond to “expression profiling by array” GSMs, and blue bars correspond to “expression profiling by high-throughput sequencing” GSMs.

The omics sciences, among the many areas of knowledge affected by this paradigm shift, have experienced significant growth in recent years. This growth is related to advances in high-throughput technologies as well as an increase in the quantity of data generated. One example is the case of the transcriptomic data found in GEO, the largest publicly available functional genomics repository [2]. This repository is composed of three types of entities (GSE, GSM, and GPL), that contains functional genomics experiments (in the form of a GEO series, GSE) recording each of the analyzed samples (within a GEO sample, GSM) in addition to the information on the experimental protocol and the technology used in the experiment (within a GEO platform, GPL). Of all the entries available in GEO, the number of those that are transcriptomic, which are primarily generated through RNA-seq and microarrays, is around 5 million. We projected that by 2030, this number will double (Fig. 1a and S1). Despite the attention on microarray data has been reduced regarding RNA-seq, both technologies have shown similar performance [3].

This amount of data has the potential to be repurposed to collect more conditions than a single experiment could replicate individually. This may lead to the generation of meta-analyses that seek to detect differentially expressed genes by analyzing previously published datasets to address specific questions associated with a phenomenon, such as a disease [4–7], adaptive stress [8], or a basal physiological process [9].

Beyond the detection of differentially expressed genes, the reuse of this data can be directed towards the generation of more complex models to understand expression patterns and the mechanisms behind them. Such is the case of inference of regulatory networks, which can be obtained using expression data [10–12]. These networks recapitulate the regulatory relationships between the available genes, offering a new interpretation of the data by presenting proposed interactions among genes along with the organization governing them, allowing the analysis of expressed genes from a systems perspective [13–15].

However, for these meta-analyses to be viable, the data must have certain characteristics, which can be found encapsulated in the FAIR (Findable, Accessible, Interoperable, Reusable) principles [16]. Specifically, the data must be of high quality and accessible, along with having a description of the technical and biological conditions for the entries to be meaningful [2, 17]. In this regard, several challenges have been reported around GEO functionality and structure, leading to the development of metadata download and annotation tools for transcriptomic data [18, 19], as well as curated databases containing subsets of GEO [20–23]. These contributions, while valuable, do not fully address the complexity of data reuse on a massive scale because they focus on small datasets corresponding to certain interests, lack of standardized protocols to functionally integrate data from diverse sources, and have a strong bias in favor of a few eukaryotic organisms, which limits the diversity of the data explored and curated.

This raises the question of whether the transcriptomic data present in GEO possesses the required attributes to be reused in the context of massive data integration, given that the possibility of integrating current data with new entries would become increasingly challenging over time. This is due to the growth rate of the number of new experiments, coupled with the progressive loss of context of the data. The latter reduces the possible reutilization of data because entries lose their usefulness given the lack of standards regarding both the metadata and the data itself [24].

Therefore, here we will analyze and describe the current state of public transcriptomic data in the GEO database, as well as technical considerations relevant for massive data integration. In addition, given the constant bias towards eukaryotic-related entries (Fig. 1b), we focus on bacteria-related data. This is an attractive starting point due to its potential for extensive use in transcriptomic analyses for biotechnological and systems biology applications. Accordingly, we focus our interest on data associated with organisms that have an annotated and sequenced genome, as these currently have the greatest potential for integration in meta-analysis.

To address these challenges, we will focus on both microarray and RNA-seq data, which comprise ~70% of the data in GEO. As mentioned above, although microarray technology is being gradually replaced by RNA-seq, we decided to include the former since research has demonstrated that this technology performs similarly to RNA-seq, except for genes with low expression levels [3]. Additionally, the processing of microarray data is associated with a relatively low computational cost in comparison to RNA-seq. Therefore, both technologies are important objects of study for the analysis and subsequent development of alternatives for mass data processing, as well as for analyzing areas of opportunity that apply to other types of experimental methodology within GEO.

In this study, we will first concentrate on the characterization of the taxonomic bias for our bacterial dataset. We will next focus on the quantitative description of limitations associated with the repository by exploring the metadata diversity both in microarray and RNA-seq datasets, and then by analyzing the raw data availability, focusing, the latter only, on microarray data since for RNA-seq this issue is partially solved by SRAtoolkit. Finally, we review methodological limitations regarding raw data processing. This analysis will focus on the microarray dataset since analysis of RNA-seq data is a widely discussed topic in the community.

### Taxonomic bias

There is an intrinsic taxonomic bias in genomic research. On one hand, there are limitations associated with the bacterial culture capability in a laboratory setting. On the other hand, there is an emphasis in some research on some model organisms. Whereas this bias is widely recognized, a quantitative description is required to understand the diversity of the data from a big data perspective. This becomes more relevant in the context of bacterial entries, where it has been observed that for both microarray and RNA-seq data, these entries constitute a minority (<3% and <2%, respectively) of the overall microarray/RNA-seq data available in GEO. Reduced sample size increases the effect of taxonomic bias, making it vital to recognize over-represented species and the number of associated entries. It is interesting to note that in the case of the bacterial dataset (~95 000 GSMs), the proportion of data associated with microarrays (48%) and RNA-seq (52%) shows the importance of the analysis for both technologies in terms of their taxonomic diversity.

In our bacterial dataset (~45 000 microarray and ~50 000 RNA-seq entries), ~48 000 (51%) were found to correspond to organisms from the superphylum Pseudomonadota (~21 000 microarrays and ~28,000 RNA-seq) that includes gram-negative bacteria such as *Escherichia coli* (Fig. 1c). Other ~22 000 (23%) entries were associated with organisms of the superphylum Bacillota (~11 000 microarrays and ~11 000 RNA-seq), which includes species such as *Bacillus subtilis* and *Staphylococcus aureus*. The remaining ~25 000 (26%) entries correspond to 23 phyla, including extremophilic, photosynthetic, and pathogenic mesophilic bacteria. However, diversity at the phylum level barely manages to recover half of the representative groups [25], leaving nine phyla of extremophilic bacteria with <250 (0.24%) GSM entries in total. These trends are in line with what is seen in genomic sequence databases, where much of the data is concentrated on a particular set of easy-to-cultivate bacteria, model organisms, or clinically relevant strains, leaving other bacterial groups understudied or simply nonexistent in the database [26, 27].

The bacterial GEO dataset demonstrates that this trend is also maintained at the species level, with ~45 000 (47%) entries concentrated in seven species out of 753 (0.92%), including *E. coli, Mycobacterium tuberculosis*, *Pseudomonas aeruginosa,* etc*.* (Fig. 1d). The remaining bacterial organisms cover a wide range of research contexts, which suggests that there is a diverse range of goals and interests surrounding bacterial data. This variety of applications can be linked to the diversity of taxonomic annotations, which provide context regarding the origin of the sample and the type of study with which it is associated. In some cases, taxonomic ambiguity is a biological consequence of the experimental design, such as new species identification, meta-transcriptomic analyses, and synthetic designs. However, to unequivocally identify the organism from which a sample came, it is necessary to rely heavily on the structure and intrinsic components of the GEO database, particularly during massive data integration. This suggests that the presence of ambiguous taxonomic annotations (e.g. “Synthetic and Chimeric”, “Environmental samples”) (Fig. 1b) presents a significant challenge to this task. These annotations are associated with the entries in the form of metadata, which are descriptive fields, and their corresponding values, facilitating the structuring and organization of the raw data within a database.

### Repository limitation: metadata

Data is only meaningful if all the context around it is equally preserved and recorded in concrete categories. This information is known as metadata, and although there is no absolute consensus on its definition throughout the literature [28], its importance in the preservation of data and its usability outside its original study framework is considered. In the case of transcriptomic data, sufficient metadata is necessary so that unified entries can be compared across the technical and biological conditions underlying the experiment.

Each of the existing entities composing GEO has a specific range of metadata that has been predetermined by the database designers. This metadata includes the record of relationships between these different entities, as well as the description of the experimental protocol, among biological, technical, and other relevant data. It is crucial to state that each metadata entry may belong to biological variables, encompassing all aspects of the study that were the subject of investigation at the experimental level.

**Figure 2:**
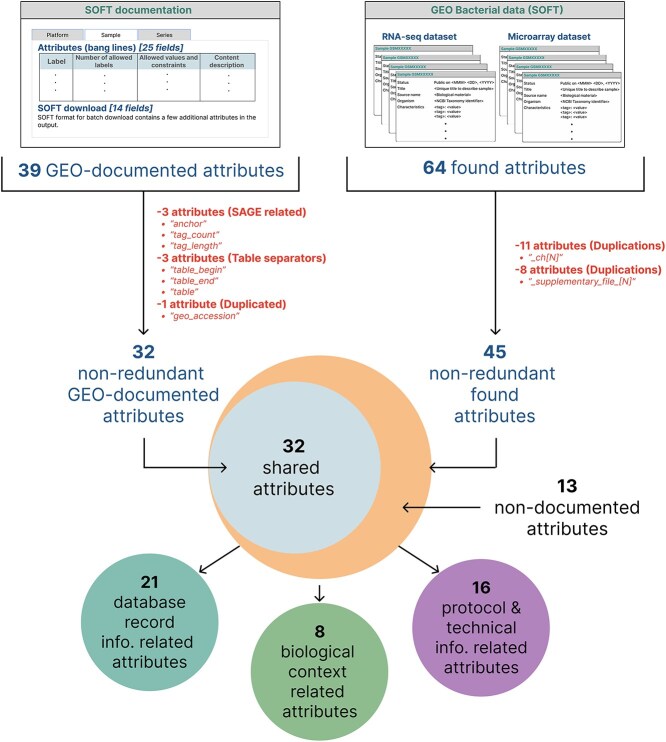
Available metadata fields in the bacteria GSM SOFT files. Venn diagrams representing the GSM SOFT attributes reported in this study. GEO only documents 39 attributes. Removing SAGE and table metadata, as well as duplicated fields, we only count 32 documented fields. Alternatively, in our bacterial microarray and RNA-seq dataset, we found a total of 64 attributes. After removing redundant metadata fields, we obtained a set of 45 metadata fields, leaving 13 nondocumented attributes. This 45 metadata can be classified according to its content (Table 1). Further information about redundant-attribute depuration is available in Fig. S1.

In GEO, these relationships are represented using the Simple Omnibus Format in Text (SOFT), a proprietary format an access to the information associated with each record in the database, including the samples and their associated metadata [29]. Although this format ceased to be used in early 2024 as a way to upload and update data in GEO, it remains the predominant approach for large-scale access and download. The metadata is presented in plain text, where each line is a field-value pair separated by “=”. In the specific case of the GSM entity, GEO documents 25 metadata fields, along with 14 fields added to the SOFT files during download [30]. Of these 39 documented metadata fields, the “geo_accession” field is duplicated on the documentation, so the number of nonredundant fields in this case is 38 (Fig. 2). It should be clarified that within these fields, three (“anchor”, “tag_count”, and “tag_length”) are exclusively for entries generated with the Serial Analysis of Gene Expression (SAGE) technology. It is important to clarify that SAGE and microarrays are the only two technologies explicitly mentioned in the documentation. These three SAGE metadata fields were discarded as they are not relevant for the study of RNA-seq and microarray entries. In addition, there are two attributes (“table_begin”, “table_end”) that serve as separators of the normalized data (identified by the “table” attribute), and therefore these three cannot be considered as a metadata field. Therefore, the official GEO documentation reports 32 (38–3 (SAGE) – 3 (table)) nonredundant metadata fields available for the GSM entity.

However, in the analyzed dataset of bacterial microarray and RNA-seq data, we observed that there were 64 GEO metadata fields, which after redundancy reduction of the channel-wise metadata (“_ch[n]”) together with those of “supplementary_file”, end up being 45 nonredundant fields (Fig. 2 and S2). This implies that 13 additional metadata fields were found compared to those reported in the SOFT GEO documentation (Table 1 and S1). The difference in quantities, together with the inconsistent use of some metadata fields (e.g. “type”), may be explained because until recently, GEO allowed the uploading of data and their respective metadata directly in SOFT format, which could have allowed the generation of unregulated and undocumented fields.

**Table 1:** Available metadata fields found in the bacterial microarray/RNA-seq dataset.

**Field**	**Source**	**Data format**	**Technology**	**Class**
characteristics	Documented by GEO^*^	Semi-structured	Both	Biological
description	Documented by GEO^*^	Unstructured	Both	Biological
growth_protocol	Documented by GEO^*^	Unstructured	Both	Biological
molecule	Documented by GEO^*^	Structured	Both	Biological
organism	Documented by GEO^*^	Structured	Both	Biological
source_name	Documented by GEO^*^	Unstructured	Both	Biological
treatment_protocol	Documented by GEO^*^	Unstructured	Both	Biological
contact_city	Documented by GEO^*^	Structured	Both	Database
contact_department	Documented by GEO^*^	Structured	Both	Database
contact_email	Documented by GEO^*^	Structured	Both	Database
contact_fax	Documented by GEO^*^	Structured	Both	Database
contact_institute	Documented by GEO^*^	Structured	Both	Database
contact_name	Documented by GEO^*^	Structured	Both	Database
contact_phone	Documented by GEO^*^	Structured	Both	Database
contact_web_link	Documented by GEO^*^	Structured	Both	Database
data_row_count	Documented by GEO^*^	Structured	Both	Database
geo_accession	Documented by GEO^*^	Structured	Both	Database
last_update_date	Documented by GEO^*^	Structured	Both	Database
status	Documented by GEO^*^	Structured	Both	Database
submission_date	Documented by GEO^*^	Structured	Both	Database
title	Documented by GEO^*^	Unstructured	Both	Database
biomaterial_provider	Documented by GEO^*^	Structured	Both	Technical
channel_count	Documented by GEO^*^	Structured	Both	Technical
data_processing	Documented by GEO^*^	Unstructured	Both	Technical
extract_protocol	Documented by GEO^*^	Unstructured	Both	Technical
hyb_protocol	Documented by GEO^*^	Unstructured	Microarray	Technical
label	Documented by GEO^*^	Structured	Microarray	Technical
label_protocol	Documented by GEO^*^	Unstructured	Microarray	Technical
platform_id	Documented by GEO^*^	Structured	Both	Technical
scan_protocol	Documented by GEO^*^	Unstructured	Microarray	Technical
supplementary_file	Documented by GEO^*^	Structured	Both	Technical
type	Documented by GEO^*^	Structured	Both	Technical
taxid	Reported in this study	Structured	Both	Biological
contact_address	Reported in this study	Structured	Both	Database
contact_country	Reported in this study	Structured	Both	Database
contact_laboratory	Reported in this study	Structured	Both	Database
contact_state	Reported in this study	Structured	Both	Database
contact_zip/postal_code	Reported in this study	Structured	Both	Database
relation	Reported in this study	Structured	Both	Database
series_id	Reported in this study	Structured	Both	Database
barcode	Reported in this study	Structured	RNA-seq	Technical
instrument_model	Reported in this study	Structured	RNA-seq	Technical
library_selection	Reported in this study	Structured	RNA-seq	Technical
library_source	Reported in this study	Structured	RNA-seq	Technical
library_strategy	Reported in this study	Structured	RNA-seq	Technical

^*^ https://www.ncbi.nlm.nih.gov/geo/info/soft.html#sample_tab

This reported metadata contains information about the record made in the database (e.g. contact data, status, and creation dates), the experimental and technical protocols (e.g. extraction protocol, hybridization protocol), and the biological context (e.g. organism, treatment protocol, growth protocol, and characteristics) associated with a sample (Table 1 and S1). Relationships to other GEO entities are also present. These fields can represent information in machine-readable format (~75%), as well as in unstructured format where the user can describe the information in natural language (~25%).

While much of the metadata seems to be in a machine-readable format, there is a bias. Of the 24 metadata fields related to technical and biological experimental variables, only three fields (“treatment_protocol”, “growth_protocol”, and “characteristics”) may contain relevant information to generate a biological context comparable between several entries, because these fields contain the description of the way the sample was handled for the transcriptomic study as well as the set of biological variables associated to the sample. The remaining fields, while relevant to the stability of the database, do not provide information about the biological/experimental context of the sample [30]. However, both “treatment_protocol” and “growth_protocol” are in an unstructured format. The lack of structured biological-related fields hinders the possibility of extracting information, as it requires the development of specialized data analyses and algorithms based on natural language processing, a subfield of artificial intelligence (AI), which requires manually curated samples to learn to identify relevant attributes.

The structure and format defined by GEO largely determine the reusability of the available metadata. This is due to the constrain produced by the presence of fields containing descriptions in natural language, decreasing the ability to extract valuable information so that the context of a sample can be compared with that of others in a standard way. These unstructured data types can be standardized using a controlled vocabulary, so that fields that normally contain long descriptions can be made machine-readable and much more explicit for submitters and curators, avoiding redundancies and ambiguities.

**Figure 3:**
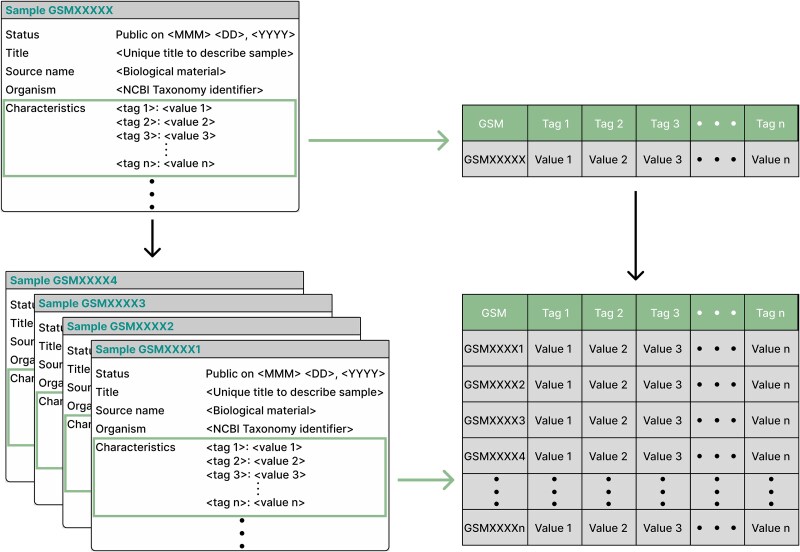
A schematic representation of the “characteristics” field available in a GSM entry. The “characteristics” field is intended to describe the attributes of the biological source in a <tag>:<value> format (https://www.ncbi.nlm.nih.gov/geo/info/soft.html#sample_tab). In principle, this format could allow users to summarize the characteristics information in a computationally tractable structure, such as a table.

Among the metadata fields available for sample entries, there is a particular field called “characteristics” which, according to the GEO documentation, should present a <tag>:<value> format recording all biological attributes describing the sample regarding its precedent study (GSE). Given a set of GSMs, this format can be reinterpreted as a 2D data array where rows are each of the GSMs and columns are each tag in the “characteristics” field. Each row contains all the values mapping to its corresponding tag (Fig. 3). This data abstraction has the potential to be a starting point for metadata exploration and subsequent curation in GEO.

Despite the above, a defined model of structured metadata is not clear. Although it should adhere to the <tag>:<value> format, it is at the discretion of each author to define how each of these tags or values should be [30]. This ambiguity leads to the question of whether the format of “characteristics” is sufficient for this field to be massively processed for biological metadata retrieval.

To be able to assess the processability potential in the “characteristics” field, we analyzed the number of putative structured metadata in the field using all ~95 000 samples of the GEO bacterial microarray/RNA-seq dataset. We define “putative structured metadata” as that metadata with <word(s)>:<word(s)> format, where the maximum number of words in both sections is limited to a discrete number. By including the adjective “putative” in the definition, it is considered that the number of words in each component of the metadata can give us an idea, without considering the word context, of how close it is to a relevant biological term, as these usually have a reduced number of words.

**Figure 4:**
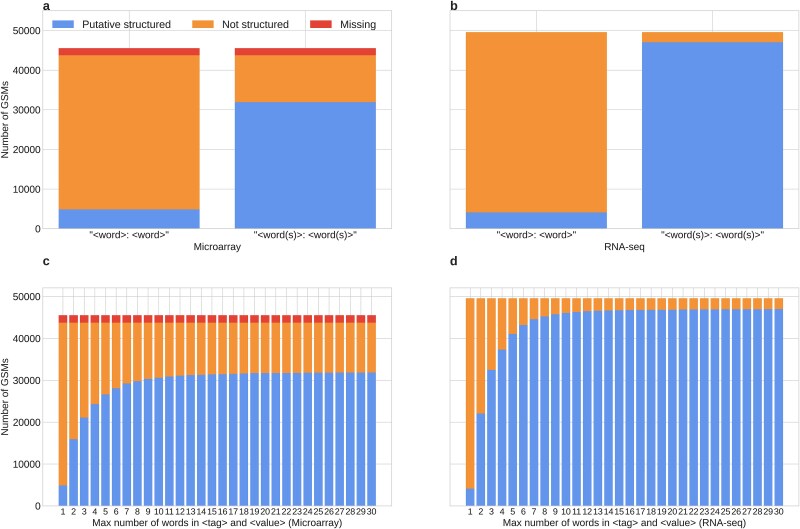
The structure of the “characteristics” field. (a) Classification of bacteria microarray GSMs according to their “characteristics” field structure. Shown in red are GSMs with no information in “characteristics”. Shown in blue are GSMs classified as “putative structured”, i.e. they follow the <tag>:<value> format. Given that this format allows tags and values to have more than one word, we show the proportion when only a single word is allowed for tag and value (left), and when any number of words is allowed (right). Shown in orange are the “not structured” GSMs. (b) Same as (a), but with bacteria RNA-seq GSMs. (c) The number of bacteria microarray GSMs classified as in (a) changes as the maximum number of words allowed for tag and value increases. The longest tag in our microarray dataset had 86 words. (d) Same as (c), but with bacteria RNA-seq GSMs. The longest tag in our RNA-seq dataset had 12 words.

For each technology included in the dataset, it was observed that, considering the strictest case (where the format is <word>:<word>), ~10% of GSMs present putative structured metadata in the “characteristics” field. On the other hand, with the more flexible interpretation, where the format is <words>:<words> and no explicit word count limit is imposed, the proportion increases in the microarray case to ~70% of GSMs, while in the RNA-seq case it increases to ~95% (Fig. 4a and b). However, even varying the stringency of the definition based on word count, at a determined point, there is no great change in the proportions of putatively structured attributes (Fig. 4c and d). It is important to consider that each GSM entity can have more than one <tag>:<value> pair in the “characteristics” field (Fig. S3), so for the putative structured classification, it is needed that all “characteristics” pairs comply the <tag>:<value> format.

The difference in the limit of possible putative structured metadata in the field “characteristics” between technologies can be partially attributed to the degree of missing values, where we observed that only microarray presents this pattern. In addition, this difference in the limit could be because RNA-seq data appears to be more stable/regulated, particularly regarding the use of special characters (Fig. S4) and the distribution of <tag>/<value> number of words (Fig. S5). This, rather than being an intrinsic product of the technology, could be linked to the time lag of the years in which each of the entries was uploaded (where microarrays have records since 2000, and RNA-seq since 2009) and to the maturity of the scientific community around biological databases. Given this, a controlled vocabulary is needed in this type of metadata, along with monitoring of the follow-up of the standards set by the GEO curators for each metadata described.

**Figure 5:**
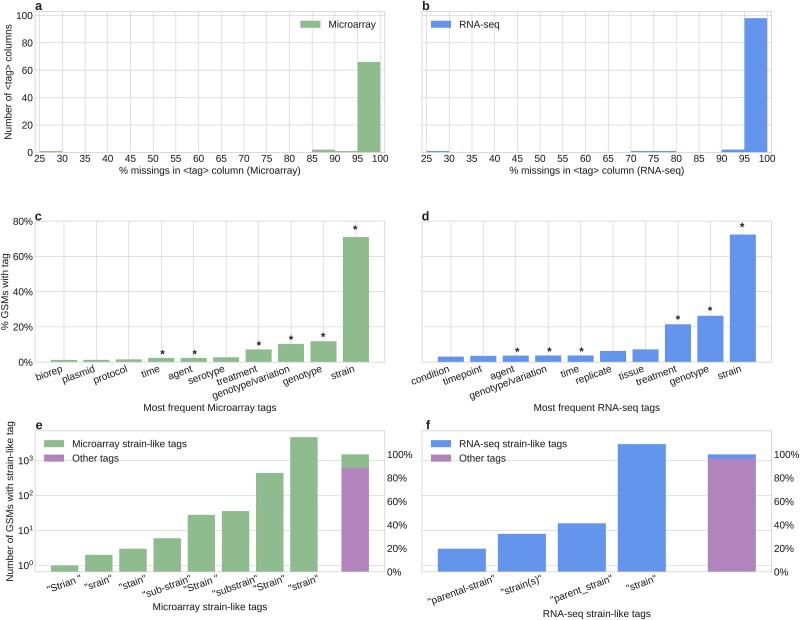
The content of the “characteristics” field. (a and b) Distribution of the percentage of missing values among the columns of a metadata table (as shown in Fig. 3) constructed using only “putative structured” <tag>:<value> pairs. (c and d) Percentage of GSMs with the top 10 most-frequent tags among the “putative structured” GSMs. An “^*^” indicates shared tags between microarray and RNA-seq data. (e and f) Distribution of the number of GSMs containing “strain-like” tags. All these tags belong to GSMs classified as “putative structured”, allowing only a single word for tag and value. Note that the y-axis is on a log_10_ scale. On the right, the bar shows the proportion of “strain-like” tags among all the tags available in the “putative structured” GSMs. In all panels, the left (green) corresponds to the bacterial microarray data, and the right (blue) corresponds to the bacterial RNA-seq dataset.

Considering that the “characteristics” field, when joined across multiple samples, can be interpreted as a data array (Fig. 3), we created a characteristics table only employing the <tag>:<value> pairs of putative structured GSMs. During the creation of this table, the union across GSMs arrays led to the presence of empty entries due to the heterogeneity of the attributes (tags) available in each entry, coupled with the lack of consensus on the biological attributes needed in a sample [30]. Most columns produced by analyzing the “characteristics” field have a high percentage of empty elements (~90%), and those with up to 30% missing elements are the minority (Fig. 5a-b). Those columns with a higher percentage of missing values may be associated with attributes unique to a few single studies.

Since there is a difference in the use of metadata between microarray and RNA-seq entries (Fig. S2), it can be assumed that the use of those minority columns with a low number of empty elements would also differ between technologies. Interestingly, it was observed that there are multiple shared tags among the top 10 most used for each technology (Fig. 5c and d and S6). However, this does not negate the fact that the number of empty elements may be increased by the redundancy of a particular tag regarding the other attributes.

This case is exemplified by the tag “strain”, which presents a redundancy in the column name, showing a considerable variety of ways to refer to the same field, and this set alone scarcely represents ~10% of the attributes available in “characteristics” (Fig. 5e and f). However, this redundancy may be due to an ambiguous interpretation of the attribute, resulting in a column name that does not initially match the column content. In the case of the columns related to “strain”, there is a large variety of strain names represented in the dataset (Fig. S7 and S8). The ambiguity sometimes caused by generic names regarding the exact taxonomy of the organism studied in each sample could be resolved by using identifiers associated with the Taxonomy database. This example shows the necessity to have standards regarding the attribute names and homogeneous units associated with the values.

Given the nature of the metadata in this dataset, it is extremely important to consider the FAIR principles as guidelines for improving the reuse of transcriptomic data. Much of the FAIRness depends on the metadata, so metadata normalization and cleaning could improve GEO stewardship. Such improvement is complex and needs to be multidimensional to address most of the already deposited data. On the GEO user side, there are tools that allow the management of certain facets of the repository (e.g. raw metadata download, normalization pipelines, etc.) that can solve the small-scale handling of microarray/RNA-seq entries (Table S2). However, these are limited by the current state of the associated metadata, where such tools cannot be used to perform large-scale reanalysis in an automated way without first dealing with the heterogeneity within GEO.

While there are already valuable efforts for metadata enhancement and mining using machine learning and natural language processing [20, 31], there are still challenges remaining, such as the fact that many strategies involving natural language processing require a high-quality corpus of data to produce statistically significant results. In this case, the GEO database itself may provide this processed corpus, giving rise to a valuable resource for mass analysis with strategies of this type. Another limitation is the a priori determination of the fields to search, and whereas mining the field “characteristics” could lead to an organic generation of consensus based on study trends for bacterial organisms [32]. This, together with the application of ontologies and controlled vocabularies for biological attributes, will be extremely useful for the re-use of transcriptomic data [33].

### Repository limitation: raw data availability

In the case that metadata could be obtained from GEO annotations, we would encounter an additional problem when trying to download and massively use these entries: the accessibility of the raw data.

This accessibility is strongly linked to the technology with which the data was obtained, so these are two parallel problems, but with different dimensions. In the case of RNA-seq, access to the raw files can be done through tools such as SRAtoolkit, which is a straightforward route (although it can be challenging to scale to a massive data download due to the nature of the .fastq files). This is not the case for microarrays, where in GEO the raw data is recorded in the form of supplementary files, which contain the original raw intensity measurements. The download and integration of these additional files pose particular challenges.

Due to the structure of GEO, not all microarray entries, although a large part, have one or more supplementary files, as this field is not mandatory. This represents a problem since these supplementary files are required for the normalization across entries with different contexts or sources so that they can be comparable (see below). Also, the merging of inputs from different sources cannot be done with normalized entries since the incorporation of different multiple processing methodologies could induce batch effects, i.e. the technical variability of samples from different sources occludes the biological variability. It is therefore vital to be able to recover these files as far as possible, to process and normalize the data according to the corresponding protocol.

**Figure 6:**
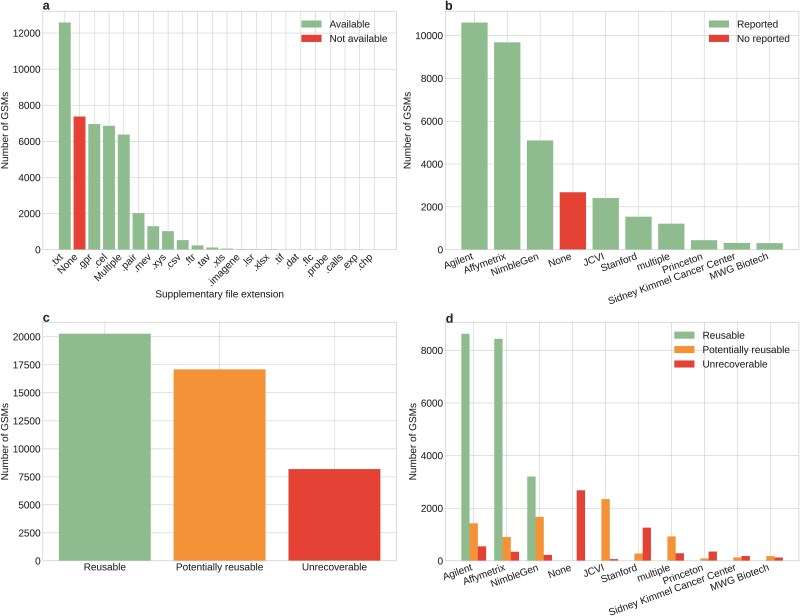
The data availability of bacteria expression profiling by array entries in GEO. (a) Distribution of the number of GSMs for each supplementary file type available for the bacterial microarray records in GEO (green). “Multiple” refers to GSMs with more than one supplementary file. Shown in red is the number of GSMs with no supplementary file provided. (b) Distribution of the number of GSMs among the 10 main manufacturers for bacterial microarrays (green). Shown in red is the number of GSMs with no manufacturer reported. (c) Reusability classification of the bacterial GSMs available in GEO. GSMs with no supplementary data, no manufacturer, only having a nonprocessable supplementary file type (nonprocessable supplementary file types include .tiff, .jpg, .xls, and .xlsx), or of noncommercial distribution, are considered “unrecoverable”. “Reusable” data corresponds to GSMs associated with GPLs of commercial or custom-commercial distribution by one of the top three manufacturers shown in (b), along with its corresponding file type (Affymetrix: .cel, Agilent: .txt, NimbleGen: .xys | .pair). The remaining GSMs are classified as “potentially reusable”. The classification assumes that the file extension is informative about its content. (d) The same classification as in panel (c), but only for the 10 main manufacturers of the bacterial GSMs available in GEO.

In the analyzed microarray data, around 7500 entries (~17%) do not have any supplementary files associated, while ~13 800 (~31%) have available files with a .txt extension (Fig. 6a). The availability of these files is only relevant when accompanied by the context of the manufacturer (descriptor of which company made the design of the microarray chip), as this allows the file can be read and processed according to the file structure. We observed that the three most common manufacturers for bacterial microarray entries in GEO are Affymetrix (~23%), Agilent (~21%), and NimbleGen (~11%) (Fig. 6b).

However, not all supplementary files available in the database have the potential to be reusable. Sometimes, users may upload all the output of their programs, including image formats that cannot be read or used for normalization. Furthermore, the GPL designs in GEO are classified as commercial, custom-commercial, and noncommercial according to how the array was manufactured [34]. Therefore, only the commercial and custom-commercial records are expected to follow a regular pattern, allowing the design of parsers for their supplementary files, while the noncommercial entries might present a less regular structure, impeding their automated processing, given the lack of a design blueprint readily available.

Moreover, each manufacturer is usually associated with a standard file format for the raw data, but there is a significant percentage of entries with incompatible file formats and manufacturer annotations. This leads to the classification of entries based on their reusability, where ~7500 (17%) entries are unrecoverable due to the absolute lack of raw data. Similarly, the categories “reusable” and “potentially reusable” exist on the premise that the availability of the supplementary files, together with a proper annotation of manufacturer and file type, can lead to the possible incorporation of the data into a massive expression table (Fig. 6c).

In the “reusable” category, the manufacturers that are represented in bacterial microarray data are Agilent, Affymetrix, and Nimblegen (note that there are manufacturer name variants, Table S3). This is because there are tools and software packages in the literature that allow automated reading and normalization of data from these manufacturers [35–37]. Other manufacturers’ data are potentially reusable, but there is no standard protocol to read those supplementary files (Fig. 6d).

### Methodological limitation: raw data processing

As in the case of accessibility, raw data processing differs strongly between microarrays and RNA-seq. In the last case, there are already exhaustive analyses in the literature on the different strategies and tools to use with raw data [38, 39], and there have been attempts to implement solutions on the different layers of medium-scale normalization [40, 41]. For microarray data, however, there is no equivalent in-depth analysis on the processing of raw data and the different levels of normalization required for mass integration. Although, as discussed previously in this work, this technology has registered a plateau in the number of new entries, the analysis does not lose any validity, particularly for the bacterial dataset, where 46% of the entries correspond to microarrays.

Microarray measurements are accompanied by subtle errors produced by human mistakes during the sample handling and the instruments’ intrinsic technical variation. When attempting to integrate microarray data on a massive scale, this technical noise may arise at different steps, from the probe to batch levels. Since this technical noise is capable of biasing data analysis, it is crucial to identify it and take it apart from the true biological variation.

Starting from the probe intensity level, background noise refers to technical variation originating from luminous contamination between adjacent spots in the array, differences in dye emissions, nonspecific binding, etc. [42]. It has been reported that background correction is important during microarray data preprocessing [43]. In this regard, distinct microarray manufacturers have developed strategies to account for this technical noise, such as the implementation of perfect match (PM) and mismatch (MM) probes on the design of Affymetrix microarrays [44]. However, a great amount of bacterial microarray data is distributed across different manufacturers (Fig. 6b), so it might not be proper to use platform-specific background correction methods, since this could induce platform-dependent batch effects. The term batch effect refers in this context to sources of variation that are not associated with a biological source, but rather a technical one, namely when it scales to clusters arbitrarily divided by technical conditions intrinsic to the laboratory that carried out experiments (see below).

Thus, integrating multiple source data into a single dataset may require a platform-agnostic preprocessing pipeline. Nevertheless, a generalized background correction algorithm is itself a challenge. It has been suggested that very simplistic modeling of background noise might indeed lead to worse results than using raw data [42]. Even more, a comparison of different normalization methods for Agilent microarray data suggested that sometimes no background correction is preferable [45]. To the best of our knowledge, there is no study addressing the effect of background correction in the integration of multiple-platform microarray data. Previous efforts only integrate data coming from a single manufacturer, perform background correction in a platform-dependent way, or avoid background correction at all (Table 2) [6, 46–49].

**Table 2:** Different approaches to processing microarray data.

**Article**	**Platforms**	**Preprocessing**	**Normalization**	**Batch correction**	**Cross-platform correction**
Reija Autio, *et al.* (2009) [46]	SMDG Affymetrix	U Affymetrix: MAS5 (background correction)	U Z-scores Housekeeping gene centering Equalized quantile normalization^*^ Weibull distribution-based normalization^*^	None	Array generation-based Gene Centering^*^
Arran K Turnbull, *et al.* (2012) [47]	DM Affymetrix Illumina	PS Affymetrix: RMA (background correction) Illumina: None	PS Affymetrix: RMA (normalization) Illumina: Quantile normalization	ComBat	Mean centering ComBat^*^ Distance Weighted Discrimination^*^ Cross-Platform Normalization
Martin Larsen *et al.* (2014) [55]	DM Affymetrix Illumina	PS Affymetrix: RMA (background correction) Illumina: NormExp (background correction)	PS Affymetrix: RMA (normalization) Illumina: None After merging: Box-Cox transformation	ComBat (visualization only) Linear model	None (relation 1:1 between experiments and platforms)
Christian Müller *et al.* (2016) [48]	SMDG Illumina	U Log_2_	U Quantile Normalization^*^	Deming regression Passing-Bablok regression Linear mixed model Polynomial regression Qspline ComBat^*^ ReplicateRUV	None (relation 1:1 between experiments and platforms)
Lavida Brooks *et al.* (2019) [6]	DM Agilent In-house (GenPix scanned)	U NormExp (background correction)	U Loess normalization (intra-array) Quantile normalization (inter-array) Log_2_ transformation	ComBat	ComBat
Valentin Junet *et al.* (2021) [49]	DM Affymetrix Illumina Agilent GeneChip	U Log_2_	U None	None	None ComBat^*^ CuBlock^*^ YuGene DBNorm UPC

**SMDG**: Same Manufacturer Different Generation

**DM**: Different Manufacturer

**PS**: Platform-Specific – The processing step was performed platform-wise

**U**: Uniform – The processing step was performed independently of the manufacturer/platform

^^*^^Best-performing methods in benchmarking studies.

A second source of technical variability in microarray data is at the sample level, i.e. differences in the fluorescence intensities across different arrays belonging to the same experiment, derived from technical variation during sample handling [50]. These differences confound the true biological variation, thus affecting data analysis and detection of differentially expressed genes [50]. Thus, several platform-specific methods have been developed to make samples comparable [36, 42, 44, 45].

These normalization methods depend on the number of channels in the array. In one-channel arrays, experimental and control samples are hybridized on different chips, whereas in two-channel arrays, the experimental and control samples are labeled with different fluorophores and then hybridized on the same chip. Different normalization methods exist to correct single-labeled and dual-labeled microarray data; however, it is common to interpret two-channel arrays as two independent one-channel arrays [51, 52].

As with background correction, a platform-agnostic normalization method is desirable when integrating multiple source datasets. Different studies have compared the performance of a variety of normalization methods; however, the results are not always directly comparable since they do not evaluate the same set of methods, or they are biased to data from a single manufacturer [35, 46, 53]. Nevertheless, quantile normalization has been proposed as a reliable method for high-throughput processing and normalization for one-channel microarrays [54]. Although this method was proposed for the normalization of Affymetrix arrays, it has been adopted for the normalization of other platforms’ data [35, 36, 42]. In addition, it has been shown to perform well in combination with other methods to remove batch effects (see below).

As the scale of integration increases further, new sources of variation emerge, such as the batch effect mentioned above, which arises when integrating data from multiple experiments carried out in different laboratories at different time points. This technical variation is known to be capable of masking true biological signals [55]. This problem has been tackled using different approaches, and several comparisons of batch effect correction methods have led to the conclusion that ComBat is robust in a variety of contexts [6, 47–49, 55, 56]. Moreover, it has been shown that ComBat performance can be improved in combination with quantile normalization at the batch level [48]. However, this imposes the interrogation of how to properly define a batch. Usually, a batch is understood as a set of samples related by the same experimental context, i.e. laboratory, personal, reagents, date of sampling, etc. However, it has been reported that “batch information is rarely explicitly provided as sample annotations by the submitters in GEO” [23]. In this sense, the GSE entity of GEO can be used only as a proxy to delimit a batch, since it is defined as “a set of related samples considered to be part of a study” [57], although it has to be considered that it is not possible to account for intra-GSE batch effects without manual curation [23].

A last source of technical variation must be considered when integrating multiple-platform microarray data. In 2006, the MicroArray Quality Control (MAQC) consortium made evident platform-dependent differences in data obtained from the same samples but hybridized on different-platform arrays, concluding that, despite leading to consistent results, different manufacturers’ datasets are not directly comparable (see Fig. 2 in [49] for a graphical inspection of the dataset) [49, 58]. These motivated studies describe “cross-platform” normalization methods as an additional data preprocessing layer, although several other authors have suggested that cross-platform differences are indeed a main component of batch effects [6, 46–49, 55]. Since these studies integrate data from a variable number of manufacturers using different preprocessing, normalization, and batch effect correction methods (Table 2), it is difficult to elucidate a clear conclusion about the best approach to integrate microarray data at a massive scale. Nevertheless, it remains that ComBat has a robust performance removing “experimental-context” and “platform-dependent” batch effects. Thus, a suitable approach would be to define a batch as a set of samples coming from a single experiment hybridized on the same platform, i.e. a GSE-GPL combination in the case of GEO.

Another, more recent interpretation of the term “cross-platform normalization” involves scaling it up even further to the integration of heterogeneous expression data, where the definition of platform refers to different transcriptomic technologies such as microarrays and RNA-seq. Several methods have been proposed to this end; however, their discussion is out of the scope of this work [52, 59–62].

Finally, another important step during data preprocessing is the mapping of platform-specific probe IDs to universal gene identifiers [63]. Some alternatives suggest using Entrez IDs or UniProt accessions to map probes to common identifiers, whereas others suggest using sequence identity to summarize probe intensities at the gene level [48, 49, 54]. The former approach may be relatively straightforward when the proper probe annotation files are supplied, such as CDF files for Affymetrix arrays, or when there are specialized packages to perform the annotation, such as those of Bioconductor [63]. On the other hand, using sequence identity is more flexible when using custom or new genome annotations [23]. However, it has been reported that inconsistency between probe annotations across different manufacturers and the fact that GEO does not guarantee the availability of the proper probe sequences decreases the comparability between datasets, further limiting data reusability [23].

## Discussion

In the last 25 years, systems and synthetic biology have largely progressed on the integration of modules dedicated to specialized functions, leading to molecular interaction networks between diverse components, whose dynamic study requires genome-wide expression data across a large set of experimental conditions. Currently, this kind of study has focused on bacterial organisms given the availability of manually curated, high-quality molecular interaction networks.

GEO is the largest publicly available functional genomics repository, where the most represented transcriptomic entries are related to microarray and RNA-seq experiments. Even though there is an astonishingly increasing number of genome-wide transcriptomic records available, to further harness its full potential, it is necessary to take into consideration the most comprehensive set of experimental conditions under any technology, which if neglected, would miss a large number of conditions that have been only explored by using older technologies.

With this in mind, the bacterial dataset is of great interest for the purpose of extending the useful life of each of the entries in an integrative context. Whereas the taxonomic bias present in the database reduces the availability and diversity of entries, ~95 000 entries were found in this dataset, making this amount of data invaluable for the application of big data strategies.

To achieve the above, each entry must have the necessary biological context described through unambiguous and comprehensive attributes, i.e. the metadata. However, it was observed that the biological metadata is unstructured in a large part of the entries in the bacterial dataset, which strongly limits the possibility of massive reanalysis, as well as restricts the interoperability of the repository. Controlled vocabularies and standards are required to generate structured metadata and consequently ensure interoperability [64], thus accelerating the reuse of GEO transcriptomic data in large-scale meta-analyses.

Whereas manual curation is a possible course of action to extract biological context and even to define controlled vocabularies, AI disruptive techniques such as LLMs (Large Language Models) and other generative AI strategies may sound like a better alternative. However, the use of generative AI techniques requires pondering some words of caution:

In a nutshell, LLMs operate by receiving an input or prompt, assessing the most probable subsequent content, and then generating an output or completion. LLMs employ neural networks with billions of parameters, trained on vast amounts of unlabeled text data through a self-supervised learning methodology [65], to construct probabilistic models during pretraining, hence enabling LLMs to acquire the ability to anticipate the subsequent word.

Hallucinations, generated content that appears factual but is fallacious, pervade generative AI and LLMs and, generally, they are a consequence of ambiguity, misinformation, bias, and knowledge gaps [66–68], which negatively affect the fourth and fifth Vs of big data, veracity, and value. Strategies to mitigate hallucinations require increasing the quantity (an enormous corpus of text data is required [68]), quality (use of structured data is preferred), and heterogeneity of training data to improve the model.

This is not possible using GEO as the availability of data is limited. An alternative is manual curation of the training data to remove bias and increase training data quality [67]. On the other hand, classical NLP techniques such as conditional random fields [69] might be used to extract biological context, as previously was done for *E. coli* [70], but they also require manually curated and structured training data. Hence, manual curation seems to be a necessary prior step to achieve the extraction of biological context.

Some types of large-scale reanalyses, such as correlation-based co-expression inference among genes, can be carried out without any biological context. However, other studies require access to biological context to leverage the full potential of transcriptomic data. The current state of GEO data and metadata does not allow the latter type of analysis, although the former is possible to some extent, but hard work remains to be done.

The construction of a multiple-source dataset requires the implementation of a platform-independent processing pipeline capable of removing technical noise and batch effects. Unfortunately, in the case of microarrays, the low availability of raw data limits the possibility of large-scale reanalysis, since this data is often missing or is present in heterogeneous formats, which depend on the manufacturer and the submitter. In addition, differences in probe names across platforms, along with the lack of straightforward access to probe sequences, restrict our ability to automatically map fluorescence intensity values to gene identifiers.

All the points described above outline the necessity of better management of the GEO database and its data, focusing on the shortcomings that compromise large-scale applications. To achieve this, consensus principles like the FAIR standards can be used to direct the efforts towards concrete objectives.

### Guidelines for FAIRification

According to our analysis, we identified that GEO does not accomplish all of the FAIR principles, mainly regarding its Interoperability and Reusability. In the following paragraphs, we provide a series of guidelines about how GEO FAIRness could be improved (in bold, the corresponding FAIR principle).

#### F4. (Meta)data are registered or indexed in a searchable resource

While data can be found matching keywords in the GEO search system accessible through the webpage, the metadata available in the GSMs is not enough to fully characterize the entries. Metadata, such as the platform technology, are only accessible through crosslinking to the corresponding GPL. This does not allow for querying directly on the GSM metadata, limiting the ability to find relevant GSMs since GSEs are often composed of several organisms or platforms. The ability to query specific GSMs could be greatly improved by having an entity-relationship scheme with a clear description of each field available in all entities.

#### A2. Metadata are accessible, even when the data are no longer available

Currently, there is no metadata field informing about the status of a GEO entry, i.e. we cannot know if a given dataset has been removed. This could mean that no GEO entry is ever removed. However, we found 25 GSMs (Table S4) deleted by GEO, as was revealed by querying each GSM on the GEO webpage, for which no metadata is available. Note that these GSMs were uploaded between 2024 and 2025, suggesting older deleted entries could have been eventually removed. To accomplish this FAIR principle, metadata about inactive entries should be marked with a specialized attribute but still be available, while data can be completely removed.

#### I1. (Meta)data use a formal, accessible, shared, and broadly applicable language for knowledge representation

The SOFT is a proprietary human-friendly and machine-readable file format. Hence, this is neither open nor supported by different programming languages. Whereas GEO also supports downloading metadata as XML files, this other format is difficult to read and interpret, and is also bulky and slow to parse. Conversely, YAML is an easy-to-read, lightweight, and quickly parsed file format supported by over 25 programming languages, making it an excellent alternative for efficiently representing and transmitting GEO metadata.

On the other hand, as discussed above, large-scale data integration is not straightforward for microarrays, as it requires access to raw supplementary files. However, this is not trivial given the heterogeneity of manufacturer file formats (Fig. 6). This limitation suggests the need to promote new microarray data access systems capable of carrying out the seamless integration of datasets independently of their manufacturer.

#### I2. (Meta)data use vocabularies that follow the FAIR principles

Submission via the SOFT file format was an ambiguous and error-prone procedure. The GEO repository still allowed SOFT submission until January 2024, despite the GEO submission policy has changed over time. Indeed, the latest RNA-seq submission templates consider metadata fields with a vocabulary controlled by drop-down menus. While these efforts are valuable for future data, they are not enough for two reasons:

On one hand, the current state of metadata cannot be overlooked. Thus, metadata standardization efforts should be promoted for all the (meta)data currently available in GEO. On the other hand, meaningful machine-readable metadata requires ontologies providing semantics to terms and their relationships. This is particularly important to describe the biological context. In this sense, more work remains to be done to develop domain-relevant ontologies, following initiatives such as BioPortal [71] that are a promising example for future improvements.

#### I3. (Meta)data include qualified references to other (meta)data

This principle states that meaningful links should be provided to describe contextual relationships among (meta)data. Whereas in GEO, normalized gene expression data are accompanied by a bunch of supplementary files (raw, processed, or normalized data), their processing relationships are poorly described, and the transition from one processing step to another is not clear. This highlights the need to implement more informative metadata, allowing for a clear distinction of each processing step’s output, although the ideal scenario proposed by MIAME and MINSEQE only considers supplying raw supplementary files. In any case, the submission of raw data supplementary files must be mandatory.

#### R1.1. (Meta)data are released with a clear and accessible data usage license

GEO imposes no restrictions on data access or distribution. Despite this, it delegates to the user the responsibility to adhere to the copyright terms of data submitters, making the license status of the data unclear for automated searches. Therefore, explicit usage rights information should be included in all GEO records.

#### R1.2. (Meta)data are associated with detailed provenance

Despite the existence of metadata fields such as “data_processing”, it is the submitter’s responsibility to provide a comprehensive description of their protocols. Thus, there is no guarantee of the quality and completeness of its content. The use of this metadata field should be mandatory, standardized, and preferably adhere to a machine-readable syntax.

#### R1.3. (Meta)data meet domain-relevant community standards

The GEO repository encourages, but does not enforce, community standards such as MIAME and MINSEQE. However, their implementation has focused on the availability of submission templates with standard-compatible fields. This underestimates the importance of structured machine-readable formats, despite the implementation of ontologies has been suggested since the original publication of MIAME. We recognize this is a tremendous challenge, as ontologies might not suffice to give a comprehensive description of an experimental protocol. However, ontologies might be the basis for the development of a specialized grammar providing an ordered and unambiguous description of these protocols, analogous to an algorithmic procedure.

The issues reported here reflect the need for a paradigm shift in the GEO philosophy. This repository holds great potential for future, massive-scale applications. Nevertheless, it has been historically left to the submitter to bear the responsibility for the quality of data and metadata. For GEO to take a step forward in the era of big data in transcriptomics, it should increase its validation requirements regarding raw data availability, as well as adopt ontology-based controlled vocabularies and machine-readable metadata syntaxes.

## Conclusion

GEO is an invaluable resource of millions of entries contributed by the community, but its useful life is mainly reduced to serving those who conducted the study. Although some reanalyses have been stored in GEO, they are limited to a mid and small-scale set of manually curated datasets. While the original goals of this repository did not include the generation of massive reanalysis, a collaborative effort between users and curators is needed to extend the potential value of its data.

Although the analyzed dataset presents characteristics typical of experiments done with bacteria, many of our conclusions can be applied to data from other types of taxonomic entities, given that the nature of the limitations is linked to the structure of GEO itself. This should be seen as an area of opportunity where all microarray/RNA-seq data, and transcriptomic data in general, can be handled under the same FAIR-centered approach.

Nevertheless, another aspect that led to the analysis of the bacterial microarray dataset is the prospect of analyzing a set of experiments, which, if categorically discarded, would miss a large number of conditions that were performed at a particular point in time when this technique was most widely used. Treating this dataset as worthy of consideration not only contributes on a technical level to biological knowledge but also aids in a paradigm shift where each element in a database can serve a greater useful function over the years, beyond the original reason for which it was made.

Further research must be conducted towards the analysis of metadata status in eukaryotic transcriptomic entries, even though there are manually curated datasets available via some tools such as recount3 [72]. Additional research can be helpful in the development of a GEO entity-relationship scheme, along with search engines, that allow looking up entries using the complete set of metadata fields. This approach can be extended to other biological repositories/databases such as ArrayExpress [73] and SRA [74].

In conclusion, the era of big data is far from being straightforward and universal in transcriptomic repositories. Even though there is a considerable volume in repositories like GEO, the velocity of aggregation of new entries is factual, and the structure of (meta)data covers a wide variety of formats, in a more integral definition, there are two Vs left behind, veracity and value. We will not undeniably be in a transcriptomic big data era until these remaining properties of the dataset are also considered, being able to truly translate the bulk of data into relevant biological insights.

**Key Points**

- Microarray and RNA-seq data exhibit a notable taxonomic bias, with the majority of entries being associated with eukaryotic organisms. On the other side, RNA-seq and microarray have a balanced proportion of bacteria-associated entries, being ~45 000 for microarray and ~50 000 for RNA-seq.
- GEO has not provided a comprehensive description of the metadata fields present in its Simple Omnibus Format in Text (SOFT), which, coupled with its flexibility for data submission, has led to the incorrect and unstructured misuse of metadata fields, which is still present to a lesser extent on RNA-seq data.
- GEO metadata problems, particularly its lack of a strict structure, lead to the impossibility of the full utilization of fields associated with biological context, complicating the massive integration and reuse of data.
- Microarray data availability shows a complex panorama at different levels, where at least 44% of the ~45 000 microarray entries associated with bacteria have the potential to be reused, given the availability of raw data.
- We discussed how far the GEO repository is from FAIRness. We detailed how GEO is failing to comply with FAIR (Findable, Accessible, Interoperable, Reusable) principles, and we provide some guidelines to address these issues.

## Supplementary Material

Supplementary_materials_bbaf560

## Data Availability

The data sets supporting the results of this article are available in the Gene Expression Omnibus repository, https://www.ncbi.nlm.nih.gov/geo/.
