# Supplementary material for: How far are we from the era of big data in transcriptomics? Lessons from the bacterial data in GEO
Source: Brief Bioinform. 2025 Oct 23;26(5):bbaf560. doi: 10.1093/bib/bbaf560 (PMC12548026; doi:10.1093/bib/bbaf560)
Supplement: Supplementary_materials_bbaf560 [file supplementary_materials_bbaf560.zip › Supplementary_materials_bbaf560_Figures_S1-S8.pdf]

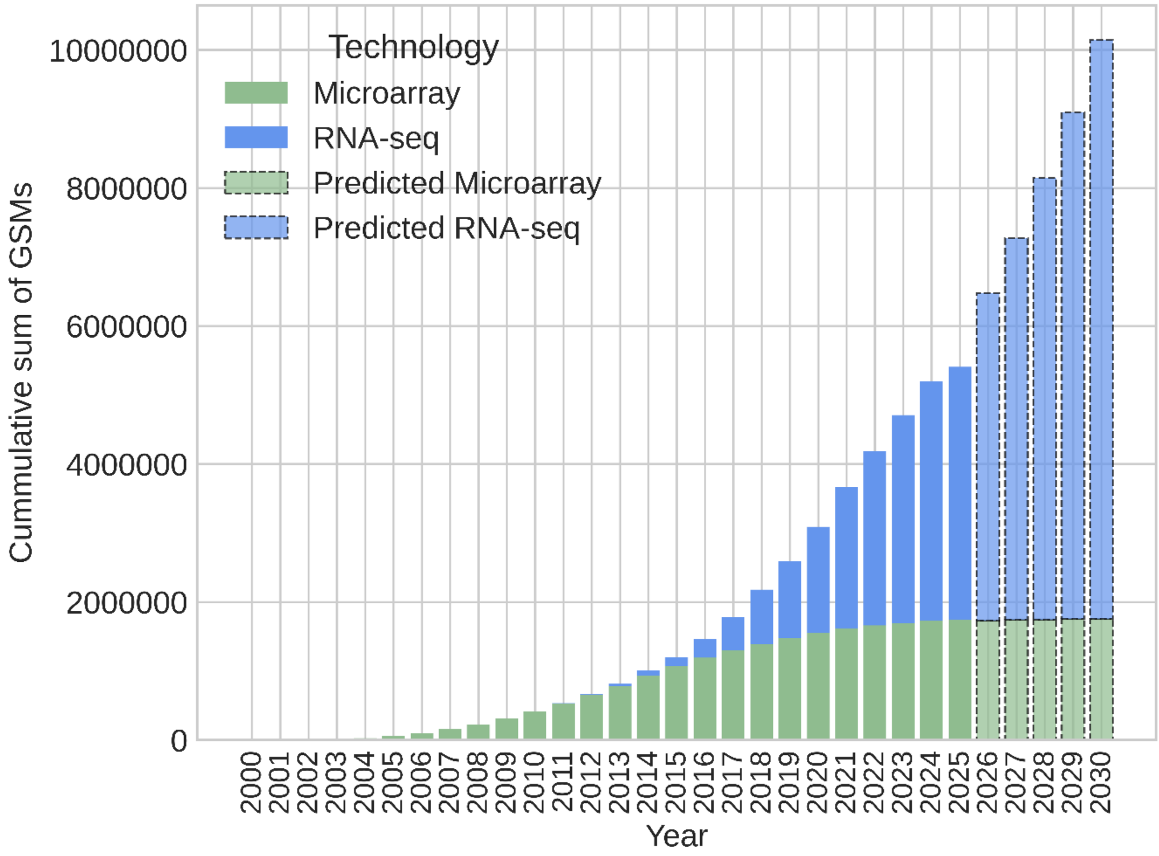

Figure S1. The amount of transcriptomic data available in GEO. The cumulative sum of the number of transcriptomic records submitted in GEO from 2000 to May 2025. In green, "Expression profiling by array"; in blue, "Expression profiling by high-throughput sequencing". Using a least squares regression, we fitted the microarray data to a logistic function (R^2 = 0.999, $y = 1763622.92/(1 + e^{-0.3314\cdot(x-2013.76)})$) and the RNA-seq data to a third-grade polynomial (R^2 = 0.900, $y = 626.35x^3 - 3768884.44x^2 + 7559354465.03x - 5053986212763.86$). We predict that the number of records will increase > 2-fold by 2030 (light-colored dotted bars).

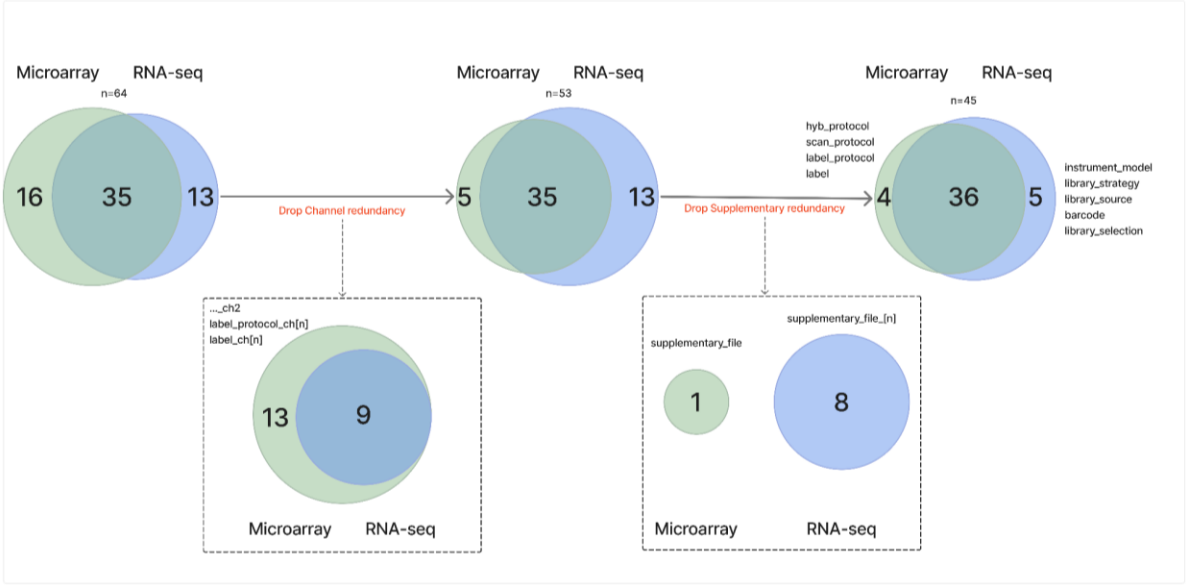

Figure S2. Redundant-attribute depuration. Our dataset starts with a set of 64 metadata attributes. From these, 16 attributes were microarray-exclusive, 13 were RNA-seq-exclusive, and 35 appeared in both datasets. Some microarray attributes are called "channel-related" because they appear once for each channel in the GSM. Please note that while microarrays can have one or two channels, RNA-seq GSMs always have one channel. To count the number of unique attributes in our dataset, we collapsed the channel-related attributes into a single field by removing the "_ch[n]" suffix. For example, the attributes 'label_ch1' and 'label_ch2' are collapsed to the 'label' field. We found a total of 11 channel-related fields. From these, 2 attributes were microarray-exclusive. After collapsing the channel-related fields, we keep 53 attributes. From these, we found 8 variants of the 'supplementary_file' attribute. All these variants were RNA-seq-exclusive. Again, we collapsed these attributes by removing the "_[n]" suffix. After this procedure, we counted 45 non-redundant fields. From these, 4 attributes were microarray-exclusive, 5 were RNA-seq-exclusive, and 36 were common to both, because the 'supplementary_file' attribute is now equal.

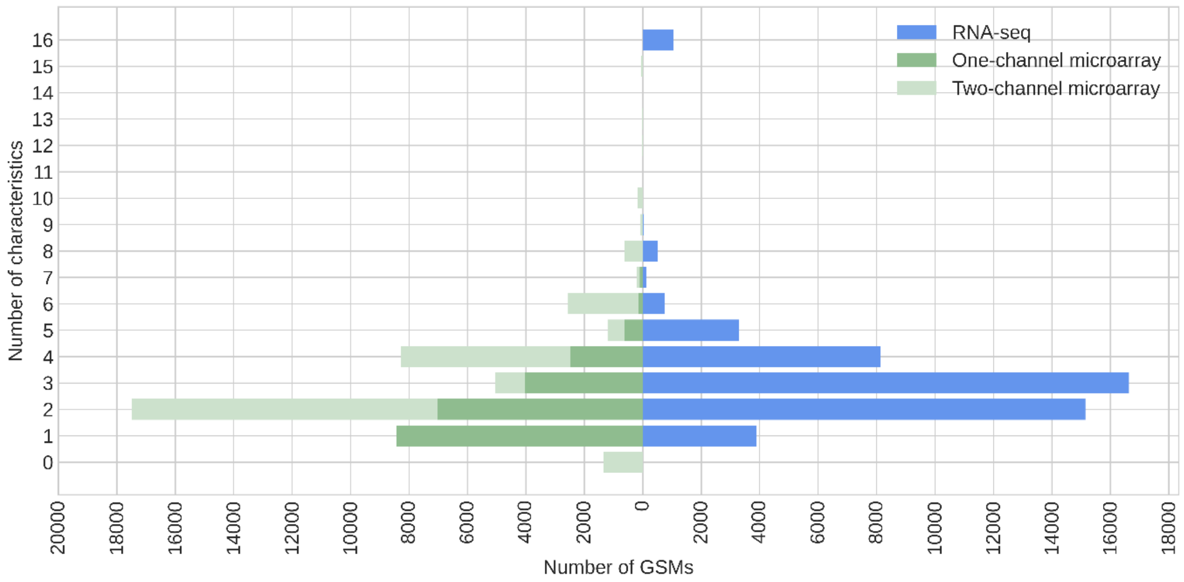

Figure S3. Frequency of the number of characteristics per GSM. Each bar represents the number of GSMs with a given number of characteristics (y-axis). Shown in green are microarray GSMs, while RNA-seq GSMs are shown in blue. Note that dark-green bars correspond to one-channel microarrays, while light-green bars are two-channel microarrays. The total number of characteristics in two-channel GSMs is the sum of the characteristics in each channel.

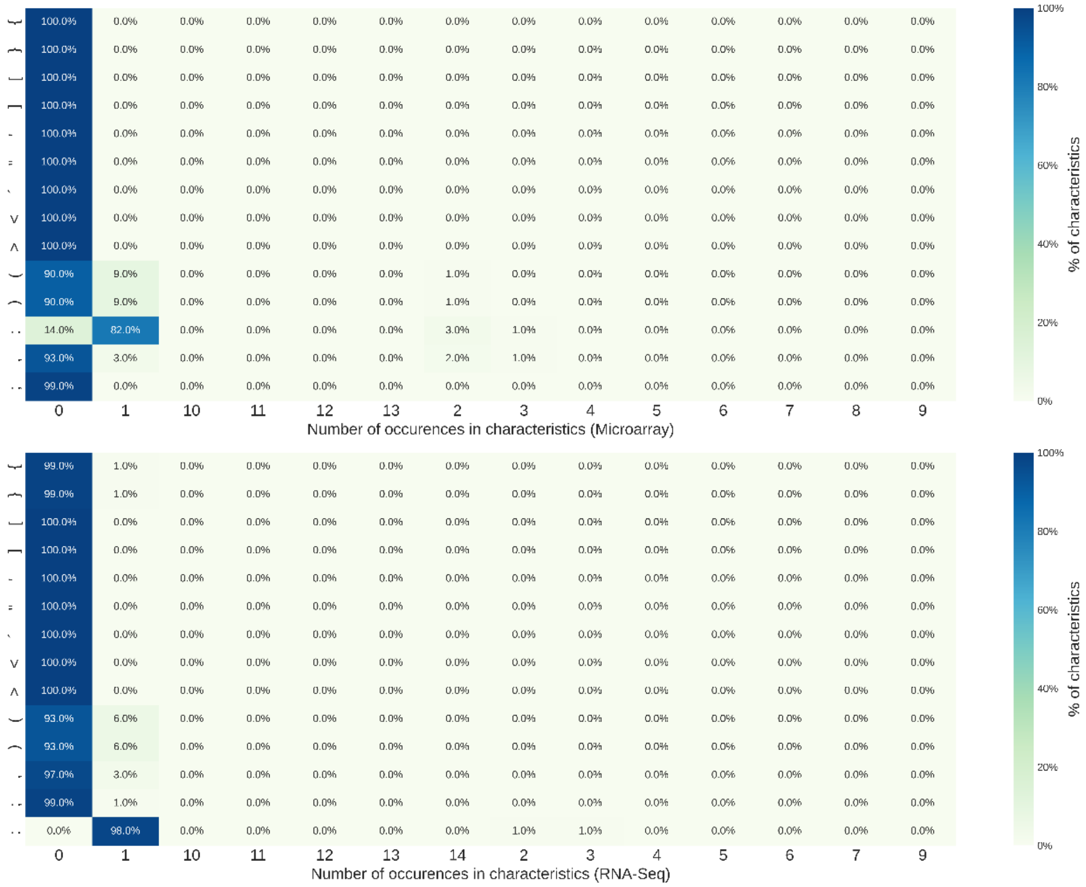

Figure S4. Distribution of the number of special characters per characteristic. On the x-axis is the number of times a given special character (each row) appears inside a characteristic (microarray data on the top, RNA-seq data on the bottom). The color is the percentage of characteristics with a given number of occurrences. Note that 82% of microarray characteristics contain a single “:” character, as expected given the <tag>: <value> format. On the other hand, 98% of RNA-seq characteristics contain a single “:” character.

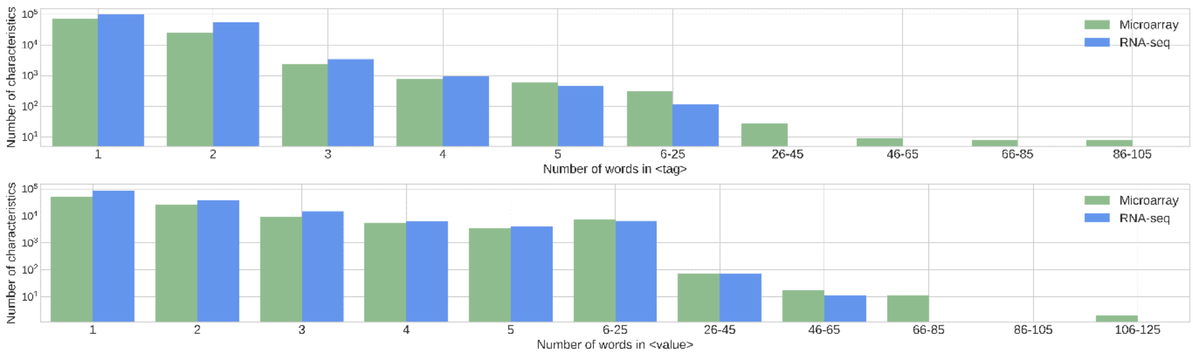

Figure S5. Distribution of the number of words in <tags> and <values>. Each bar is the number of characteristics (microarrays in green, RNA-seq in blue), with a given number of words in the <tag> (top) and <value> (bottom). Characteristics <tags> and <values> with more than 6 words are grouped in 20-sized bins. Note that the y-axis is on a log scale.

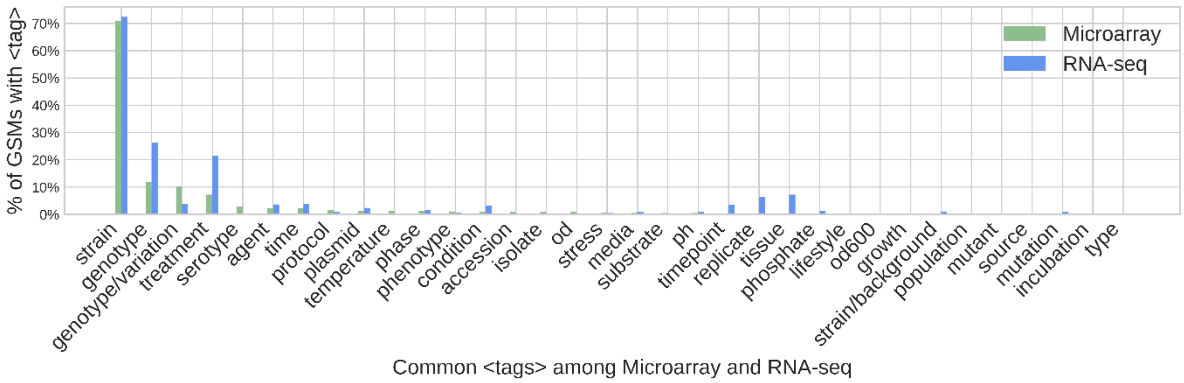

Figure S6. Percentage of GSMs with <tags> shared between Microarray and RNA-seq. Each bar is the percentage of “Putative structured” GSMs with a given <tag> (x-axis) among its characteristics. In green, microarray GSMs; in blue, RNA-seq GSMs.

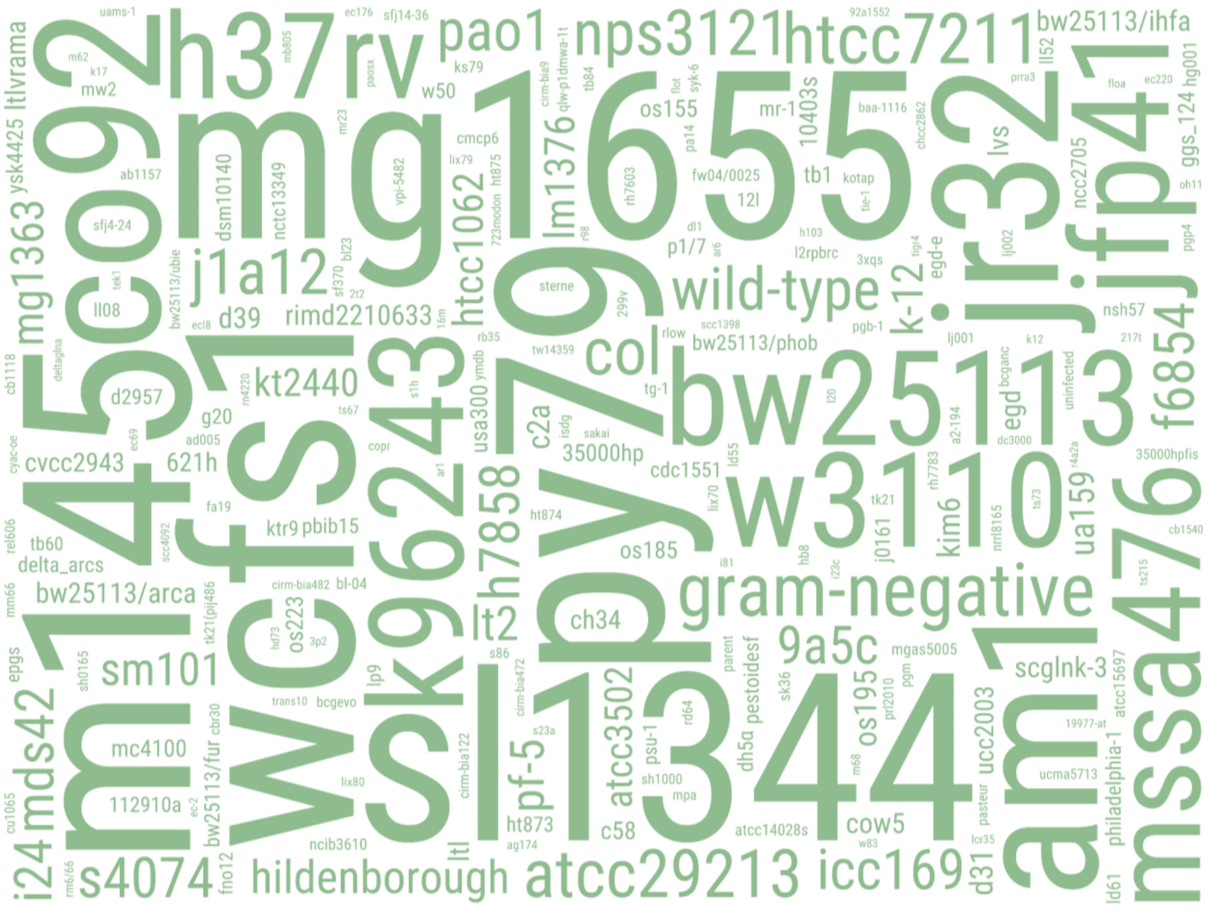

Figure S7. Microarray <strain> values word cloud plot. The plot shows the freedom in the vocabulary used in the values of the microarray “strain-like” tags.

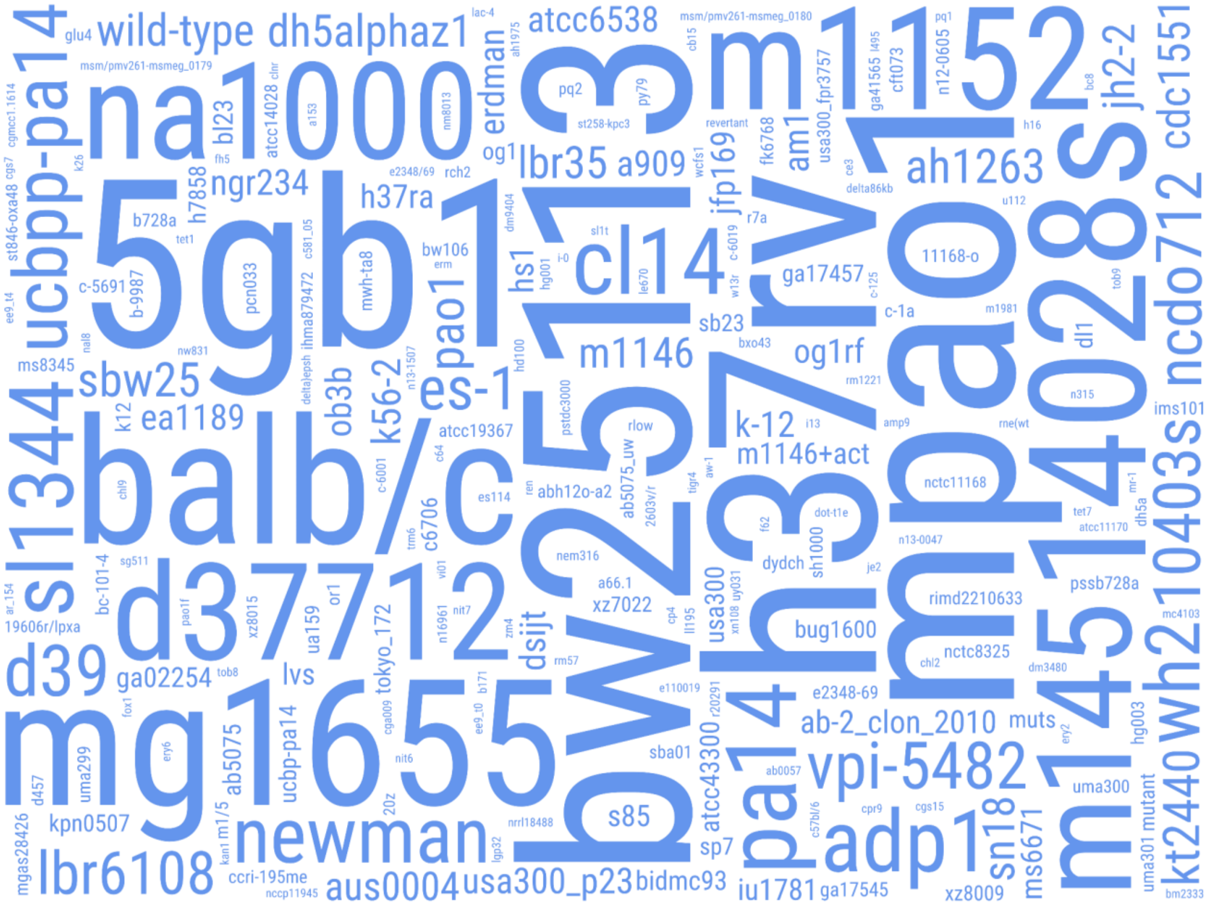

Figure S8. RNA-seq <strain> values word cloud plot. The plot shows the freedom in the vocabulary used in the values of the RNA-seq “strain-like” tags.
